# Supplementary material for: Cohesin architecture and clustering in vivo
Source: eLife. 2021 Feb 17;10:e62243. doi: 10.7554/eLife.62243 (PMC7932697; doi:10.7554/eLife.62243)
Supplement: Supplementary file 2. [file elife-62243-supp2.docx]

**Supplementary File 2: Oligonucleotides used for RT-qPCR.**

***Arm TRM1 CAR***

VG641/VG642 (AAAGAAGCAGGGGTAGAGAAGC / ATCAGCAGCGGTGATTACAC)

VG625/VG626 (CCAGCCAGATATTATGGGCAAG / TCCTAGACCTGGTGGAAAAAGC)

VG627/VG628 (CTTATAGTTCCCAAGGCATCCC / CCAAACTCGTTGTTCTCGATCC)

VG629/VG630 (TCTTCGTGCGCGAGGATATG / CGAACATTTCCGGACAATTGC)

VG633/VG634 (CCAATCGTATAACGGAGCATTGG / TGGTGCCAGAAGATATCAACG)

VG637/VG638 (GCGCGATACCATTCAGAACATC / TTAAAGTGGGCCCAAGACCAG)

VG639/VG640 (GGGCATCACCTTTTCGTAAGC / TGATCCACCTGTCATTTCGC)

VG643/VG644 (ACCCTTCTGTTCCAGTTTGC / GTTGCCTCCGGAGCAAATTC)

***Arm NUP170 CAR***

NUP170-1F/1R (CAATTCTAACGGCAGGGTTATTG / CGACTTCGCTTTCCTCGTATAA)

NUP170-3F/3R (GCATGTGAAGTTGCTGGTATTC / ACAGCTCACTTGTGCTCAATA)

NUP170-4F/4R (CGTTAAGAACAGCGGCAATAAT / ACACGTACATTACCCTGCTATC)

NUP170-4aF/4aR (CGAACAGGTCAGAGAGA / ACAGTAAGGTGGAGTTAATG)

NUP170-5F/5R (GTAAGGTCAGCAGGAACTAGATATT / GCTGACAGGTTCAAGAATAGGA)

NUP170-6F/6R (TTCTGTTGACCAGCTCTCTTG / GCTATTAGAGGCTTGCCAGAA)

NUP170-7F/7R (GGCACGGACAAATGTGAATAAG / TCGTCGTTTGAAGGGTGAAA)

***Arm TOD6 CAR***

TOD6-1F/1R (GGCCTATCTTGTCTCATCATCTT / ATCGCATCGCATCTCATCTC)

TOD6-2F/2R (TTGGGCTGGAAGGAGATTG / TCGCATACACGCCGAATAG)

TOD6-3F/3R (CAAGACAGACCACGCAAATG / CTCTAGACCACGGGTGTTTATT)

TOD6-4F/4R (ATACCCATCGCCGCTTATTC / GATGATGATGAGGATGGGAAGAG)

TOD6-5F/5R (TGCTCACTACTTCTTCTTCTTCTT / ACTTAGAACCCACCGCATTAG)

TOD6-6F/6R (CTCTGTATCCTCTCTCTCCGTTAG / GGCGGGAGAATGTCTTGTATT)

TOD6-7F/7R (GATTAGAGGACCGGATGATGTT / CAGCGAGGAGTTGAAGGTTAG)

***Pericentric CARC1***

TE367/TE368 (AAAGGTGCCCCAAGAAAAGG / AGCACTTTACTCGCTTGTGG)

TE310/TE311 (TAAAGCATTGACGCCAGAGC / AAGTACGCGTACGAAGCATC)

TE308/TE309 (TCCTGGAATGGAGACCGTTTTC / AGCCGACAAATTTCGTGCAC)

TE373/TE374 (ACTTTGGTTTTCCGGTGTGC / CCAGCGATGAGATGCGAAAAG)

TE377/TE378 (TCGCTTTTCGCATCTCATCG / AGCGGGCGGGTTATAAATAAC)

TE533/TE534 (ACCTTCTACTTCCATGCCGTTG / TGCGTGCCGATGTAGAATTG)

***CEN* primers**

***CEN4* flanking primers**

BR463/BR464 (CATGATTCGCCGGGTAAATA / GCACTAGCCAATTTAGCACTTC)

BR465/BR466 (AAAATGCCGAGGCTTTCATA / TGACGATAAAACCGGAAGGA)

***CEN14* flanking primers**

TE442/TE443 (TTAAAGCGGCTGAGTATGGC / TTTCCTCCATTGCTCTCTACGG)

TE446/TE447 (ACTAAAAGTGCCCCAAACGG / AGGAGCAGGGTAGCATAAACC)
